# Cardiovascular hemodynamic effects of Red Bull® Energy Drink during prolonged, simulated, monotonous driving

**DOI:** 10.1186/2193-1801-2-215

**Published:** 2013-05-09

**Authors:** Takehiro Yamakoshi, Kenta Matsumura, Shota Hanaki, Peter Rolfe

**Affiliations:** School of Mechanical Engineering, College of Science and Engineering, Kanazawa University, Kakuma-machi, Kanazawa, Ishikawa, 920-1192 Japan; Graduate School of Natural Science and Technology, Kanazawa University, Kakuma-machi, Kanazawa, Ishikawa, 920-1192 Japan; Department of Automatic Measurement and Control, Harbin Institute of Technology, 92 West Dazhi Street, Nan Gang District, Harbin, 150001 China; Oxford BioHorizons Ltd, 31-33 Albion Place, Maidstone, ME14 5DZ UK

**Keywords:** Energy drink, Driving, Hemodynamic reactivity, Sleepiness

## Abstract

**Purpose:**

The purpose of this study was to investigate the cardiovascular hemodynamic effects of Red Bull® Energy Drink during prolonged, simulated, monotonous driving.

**Methods:**

This was a double-blind, within-subjects-design, crossover study. Twelve healthy volunteers (21.7 ± 0.8 years old) experienced each of three conditions at various times: 1) consumption of Red Bull® Energy Drink; 2) consumption of placebo-controlled drink; and 3) no test drink. All subjects undertook 90-min periods of simulated monotonous driving, during which physiological measurements were made. The variables recorded were cardiovascular indices, *i.e.,* mean blood pressure (MBP), cardiac output (CO), electrocardiogram RR interval (RR), total peripheral-vascular resistance (TPR: *=* MBP/CO), and normalized pulse volume (NPV). Additional parameters were the standard deviation of lateral position, *i.e.,* the weaving of the car, and subjective rating of sleepiness.

**Results:**

CO, RR, and TPR during the monotonous task were significantly different in those consuming the energy drink as compared with those receiving the placebo and as compared with no drink values. The energy drink elicited a cardiac-dominant reaction pattern, while the other conditions demonstrated the vascular-dominant reaction pattern typically observed in monotonous driving tasks. The observed differences indicate the cardiovascular system being more aroused with the energy drink.

**Conclusion:**

The effects of Red Bull® Energy Drink were reflected in cardiovascular hemodynamic phenomena especially to the heart function, and we conclude that consumption of this drink before long-distance driving in non-sleepy drivers could facilitate more physiologically active, and possibly safer, driving.

## Introduction

Driving while drowsy is a major cause of serious traffic accidents in industrial nations (Philip et al. 2001). Causes of driver sleepiness include sleep restriction, sleep disorders, circadian factors, and driving-related factors such as monotonous driving and low traffic density. A motorway is a prime example of a monotonous driving environment, and some studies estimate that sleepiness accounts for about 20% of all motor vehicle accidents on such roads (Maycock 1996; Horne and Reyner 1995).

To prevent sleep-related traffic accidents on motorways, public campaigns recommend scheduled breaks, preferably involving a nap, between driving sessions. Although such breaks are the most effective means of reducing traffic accidents (Reyner et al. 2010), drivers usually take other countermeasures against sleepiness during or before driving, in particular using strategies such as opening the window and listening to music while driving. However, a recent study reported that these strategies cannot be recommended as sole countermeasures against driver sleepiness (Schwarz et al. 2012). As a commonly used alternative, consumption of caffeinated beverages, *e.g.,* coffee, tea, colas, and energy drinks, could be useful. This countermeasure has been proven effective, particularly when driving in the early morning, at night, or when sleep restricted (Biggs et al. 2007; Philip et al. 2006; Reyner and Horne 2000,1997).

Energy drinks have recently become popular worldwide. From a global perspective, one of the most popular energy drinks appears to be *Red Bull® Energy Drink* (RBED). The RBED sold in Japan contains several ingredients including caffeine, arginine, B vitamins, and inositol, a combination that differs slightly from that in the original version used in all other studies to date. The original version has been demonstrated to produce positive effects on cognitive performance, fatigue, attention, and driving performance in prolonged driving (Mets et al. 2011; Gershon et al. 2009; Reyner and Horne 2002; Horne and Reyner 2001) and extended endurance and improved exercise performance (Ivy et al. 2009), in the absence of ergogenic benefit for women athletes engaging in sprint-based exercise (Astorino et al. 2012). In addition, in laboratory tests of mental performance, RBED improved reaction time, subjective alertness, concentration, and memory (Warburton et al. 2001; Alford et al. 2001). In terms of the positive effects on driving performance, validation tests were based on the measurement of performance indices, *e.g.,* the number of lateral lane crossings, standard deviation of lateral position (*i.e.*, weaving of the car), and reaction time; questionnaires on subjective sleepiness; and physiological indicators of sleepiness (electroencephalography) and fatigue (heart rate variability). This information has been derived from driving simulator studies (Mets et al. 2011; Gershon et al. 2009; Reyner and Horne 2002; Horne and Reyner 2001).

However, despite such findings, further studies are necessary to validate the positive effects of RBED. In particular, to the best of our knowledge, there are no published studies concerning the detailed cardiovascular hemodynamic effects of RBED during prolonged motorway driving. Such studies are desirable because cardiovascular reactivity is known for reflecting arousal (Lang et al. 1997). In the present study we therefore focused on hemodynamic reactivity, assessed during an experiment using a driving simulator. An experimental paradigm was developed in which 500 ml of placebo-controlled drink or the same volume of RBED mixed with orange juice was consumed, followed by 90 min of monotonous driving. In a third condition, the same subjects drove for 90 min without having consumed any test beverage. Based on the previous research discussed above, we hypothesized that, compared with placebo-control or no drink, RBED would have a significant effect on cardiovascular variables.

## Subjects and methods

This study was a double-blind, placebo-controlled, crossover trial with a three-stage, within-subjects design. The study safeguards and protocols were approved by the ethics commission of the Faculty of Medicine of Kanazawa University on June 2011 (No. 201), and the study was performed in accordance with the ethical standards laid down in the 1964 Declaration of Helsinki and its later amendments. All subjects agreed to take part in the study voluntarily and signed an informed consent statement before participating.

### Subjects

Twelve healthy adult men, with a mean age of 21.7 ± 0.8 years and body weight of 63.2 ± 4.1 kg participated in the study. Subjects were regular drivers (more than 5,000 km/year), had been in the possession of a driver license for at least one year, were non-smokers, and had regular sleeping hours. The Japanese version of the Epworth sleepiness scale (JESS) was administered to assess general levels of daytime sleepiness (Takegami et al. 2009). Subjects with JESS scores exceeding 10 were excluded from participation (Mets et al. 2011). Other inclusion criteria were moderate caffeine consumption (one to three glasses of caffeine-containing beverages per day) and infrequent energy drink consumption (less than one drink per month). Subjects abstained from alcohol consumption from 24 h before the start of the test day.

### Experimental design

The study was comprised of one training day and three test days for each subject. On the training day, participants were screened and familiarized with the test procedures using a driving simulator, which is described below. Participants were assigned at random to a protocol in which each of the following conditions including a 2- or 3-day reversal period were applied in the order shown in Table 1: (1) consumption of RBED (*RB*), (2) consumption of placebo-control drink (*Cont*), and (3) no test drink consumed (no drink; *ND*). The order of the conditions was balanced across subjects.

**Table 1:** **Summary of the study design**

Group	Number of subjects	1^st^stage	Reversal period	2^nd^stage	Reversal period	3^rd^stage
A	2	*ND*	2 or 3 days	*Cont*	2 or 3 days	*RB*
B	2	*ND*		*RB*		*Cont*
C	2	*Cont*		*ND*		*RB*
D	2	*Cont*		*RB*		*ND*
E	2	*RB*		*ND*		*Cont*
F	2	*RB*		*Cont*		*ND*

Note: The study was a double-blind, placebo-controlled, 2 × 6 crossover trial with a three-stage, within-subjects design.

***Note:****ND = no test drink administered, Cont = placebo-controlled drink, RB = Red Bull® Energy Drink.*

On each of the test days, after checking all abovementioned criteria, the four stages of the experiment were performed in the following order with subjects sitting in a quiet, temperature-controlled, driving simulator: (a) sensors were attached in the appropriate positions, which took at least 15 min; (b) a rest period of 10 min; (c) the drink was consumed over a period of 5 min; and (d) a simulated monotonous driving period of 90 min. Each test was performed over a 120-min period. In order to simulate a real monotonous driving situation, each subject was previously informed that they had to continue driving safely, as though they were actually driving, that they should maintain a speed of 80~120 km/h, and that they should drive within a specified lane. Test sessions were performed either in the morning (10:00-12:00 AM) or in the afternoon (13:00-15:00 PM) in a balanced manner. Each subject started each test day at the same time. In addition, for each of the test sessions, subjects were allowed to have a meal 2 h prior to the experiment so that the food was not likely to affect the subject’s sleepiness.

### Test drinks

The test drinks were: 1) 250 ml of RBED mixed with 250 ml orange juice (Tropicana 100% Fruit Orange, Kirin Beverage Company Ltd., Tokyo) or 2) 250 ml orange juice mixed with 250 ml sparkling water (placebo-control drink), administered before driving. Each 250-ml RBED (Red Bull® Energy Drink 250 ml, Red Bull Japan Ltd., Tokyo, Japan) contains 10.7 g carbohydrate, 80 mg caffeine, 120 mg arginine (amino acids), 80 mg sodium, and 7 mg inositol and vitamins (niacin, pantothenic acid, vitamin B6, vitamin B2, and vitamin B12). It should be noted that taurine is not included in the Japanese drink. The placebo-control drink was adjusted using Warburton’s method (Warburton et al. 2001) to ensure it had a similar taste and color to the RBED mixed with orange juice. Drinks were consumed within 5 min before driving. After the study was completed and data were analyzed, the treatment code was revealed by a person at Kanazawa University who had not participated in the rest of the study.

### Measurement quantities and apparatus

Driving tests were performed using a driving simulator, which had previously been constructed at Kanazawa University and validated in terms of usefulness as a simulator (Yamakoshi et al. 2009b; Yamakoshi et al. 2009a). The simulator consists of an adjustable car seat, steering wheel, gear lever, clutch, brake, and accelerator pedals for vehicle control. The system generates realistic monotonous roadway scenery, which is projected on a 1219 × 1626-mm screen, placed 1.3 m in front of the midpoint between the driver’s eyes. The screen provided the driver with a 65° horizontal field of view. Auditory feedback is provided by speakers and includes engine sound. In addition, the road condition is transmitted through the steering wheel by mechanical vibration.

We measured the following cardiovascular variables during the experiment: systolic, mean, and diastolic blood pressure (SBP, MBP, and DBP, respectively) in the proximal phalanx of the left index finger; cardiac output (CO), determined using admittance cardiography triggered by the electrocardiogram RR interval (RR); total peripheral-vascular resistance (TPR *= MBP/CO*); and normalized pulse volume (NPV) as an indication of local peripheral vascular resistance. These variables were all acquired in a beat-by-beat manner. The BP and CO monitoring systems were developed as experimental instruments and have been described fully elsewhere (Yamakoshi 2004; Yamakoshi et al. 1980; Ito et al. 1976) The BP system, utilizing the volume-compensation principle (Yamakoshi et al. 1980), is capable of measuring instantaneous BP in the finger, and the admittance cardiograph (Ito et al. 1976) provides an instantaneous indication of CO. The finger photo-plethysmograph (MPN1001, Medisens Co. Ltd., Tokyo, Japan) consists of a near-infrared light source and a photosensor, which were placed on opposite sides of the distal part of the basal phalanx of the left middle finger. NPV was obtained from the DC and AC (pulsatile) components of the finger photo-plethysmograph signal. This measure has been proposed as a valid index of *α*-adrenergic sympathetic activity in the finger arteriolar vessels (Matsumura and Yamakoshi 2013; Lee et al. 2013; Sawada et al. 2001). A logarithmic transformation was applied to the NPV values (= *ln*NPV) to normalize the distribution.

Driving performance was measured by a single-axis accelerometer (FAS-A, Microstrain Inc., Williston, VT, USA) attached to the center of the steering wheel: we adopted the standard deviation of the steering-wheel reversal rate (SRR) (Oron-Gilad et al. 2008) as an index of driving performance.

In this experiment the level of sleepiness, as estimated from verbal answers to a questionnaire read to subjects by an investigator at 10-min intervals during the driving task, was also measured. Subjects reported their perceived sleepiness on a 9-level scale from Level-1 (very alert) to Level-9 (very sleepy, an effort to stay awake, fighting sleep); this is referred to as the Japanese version of the Karolinska sleepiness scale (KSS-J) (Kaida et al. 2006).

### Data analysis

The data for each condition (*RB*, *Cont,* and *ND*) and period (0–30, 30–60, and 60–90 min) were compared statistically by means of two-way repeated-measures analysis of variance (ANOVAs). The Greenhouse-Geisser correction was applied to the degree of freedom where appropriate. Bonferroni tests for post-hoc comparison were used with a significance level of 5%. To exclude individual differences in absolute value, the delta change (Δ) in reactivity was calculated by subtracting averaged rest period (5-min) values from each value, and then these values were averaged over 30 min. Statistical analyses were performed using IBM SPSS Statistics 21 for Windows (IBM Inc., Tokyo, Japan).

## Results

**Figure 1:**
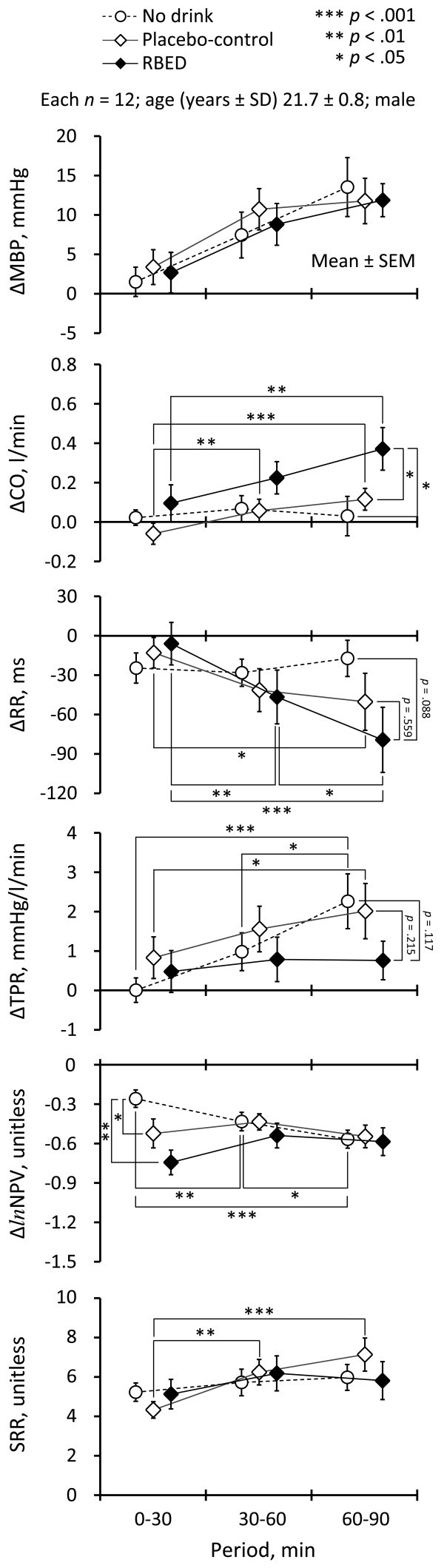
**Summarized results of the effects of condition (RBED, placebo-control, and no drink) and period (0–30, 30–60, and 60–90 min) on cardiovascular variables and SRR.** Note: *RBED =* Red Bull® Energy Drink; *SD =* standard deviation; *SEM=* standard error of the mean; *MBP* = mean blood pressure; *CO* = cardiac output; *RR* = electrocardiogram RR interval; *TPR* = total peripheral-vascular resistance; *lnNPV* = *ln*-normalized pulse volume; *SRR* = standard deviation of the steering-wheel reversal rate.

**Figure 2:**
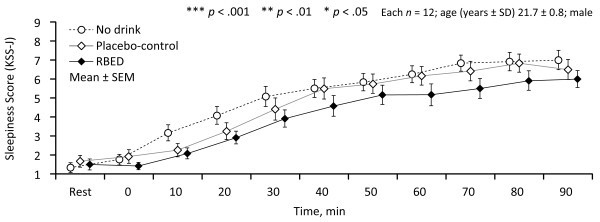
**Summarized results of the effects of condition (RBED, placebo-control, and no drink) and period (0–30, 30–60 and 60–90 min) on KSS-J.** Note: *KSS-J* = Japanese version of the Karolinska sleepiness scale.

Summarized results of condition (*RB*, *Cont,* and *ND*) in each of the three periods (0–30, 30–60, and 60–90 min) in terms of cardiovascular variables and SRR, and KSS-J are shown in Figure 1 and Figure 2, respectively. The results of a series of separate ANOVAs are summarized in Table 2.

**Table 2:** **Summary of results of two-way repeated-measure analysis of variance**

Measures		Main effect						Interaction		
		Condition			Period			Condition × Period		
		***F***_2, 22_	***p***		***F***_2, 22_	***p***		***F***_4, 44_	***p***	
Hemodynamics										
	MBP	0.08	n.s.	.007	27.2	<.001	.712	0.83	n.s.	.070
Cardiac										
	CO	3.19	n.s.	.225	13.8	<.001	.557	2.70	<.05	.197
	RR	0.49	n.s.	.043	11.6	<.001	.513	7.22	<.001	.396
Vascular										
	TPR	0.95	n.s.	.079	12.1	<.001	.524	3.13	<.05	.221
	*ln*NPV	2.81	n.s.	.203	2.10	n.s.	.160	7.76	<.001	.414
Performance										
	SRR	0.19	n.s.	.017	12.3	<.001	.528	0.84	<.01	.259
		***F***_**10, 100**_	***p***		***F***_**10, 110**_	***p***		***F***_**20, 220**_	***p***	
Subjective										
	KSS-J	2.42	n.s.	.180	54.9	<.001	.833	1.24	n.s.	.102

***Note:****MBP = mean blood pressure, CO = cardiac output, RR = electrocardiogram RR interval, TPR = total peripheral-vascular resistance, lnNPV = ln-normalized pulse volume, SRR = standard deviation of the steering-wheel reversal rate, KSS-J = Japanese version of the Karolinska sleepiness scale.*

*Each condition n = 12.*

### Mean blood pressure: MBP

We observed a significant main effect of period: MBP gradually increased over time for all conditions.

### Cardiac output: CO

The interaction between condition and period was significant. There was a significant main effect of period: CO gradually increased over time in *RB* and *Cont*. Among the conditions, post-hoc tests were significant for *RB* vs. *ND* (*p* < .05) and *RB* vs. *Cont* (*p* < .05) for the last 60–90 period; RBED increased CO significantly more than the other two conditions.

### Electrocardiogram RR interval: RR

The interaction between condition and period was significant. There was a significant main effect of period: RR gradually decreased over time in *RB* and *Cont*. Among the conditions, post-hoc tests showed RBED tended to reduce the RR for the last 60–90 min period, but this was not significant (*RB* vs. *ND*, *p* = .088).

### Total peripheral-vascular resistance: TPR

We observed a significant main effect of period: TPR gradually increased over time in *ND* and *Cont*. Among the conditions, post-hoc tests showed that TPR tended to be smaller for RBED than for placebo-control drink or no drink for the last 60–90 period, but this trend did not reach significance (*RB* vs. *ND*, *p* = .117; *RB* vs. *Cont*, *p* = .215). Unlike *ND* and *Cont*, RBED did not affect TPR.

### Normalized pulse volume: NPV

The interaction between condition and period was significant. Among the conditions, post-hoc tests were significant for *RB* vs. *ND* (*p* < .01) and *Cont* vs. *ND* (*p* < .05) for the first 0–30 min period; RBED and placebo-control drink reduced the NPV (*i.e.*, caused vasoconstriction) in the first period.

### Standard deviation of the steering-wheel reversal rate: SRR

The interaction between condition and period was significant. There was a significant main effect of period: SRR gradually increased in *Cont*.

### Japanese version of the Karolinska sleepiness scale: KSS-J

We observed a significant main effect for period: KSS-J gradually increased over time with all conditions.

## Discussion

In previous studies, the effects of Red Bull® Energy Drink, RBED, were assessed in terms of subjective driving quality and driving performance (Mets et al. 2011; Gershon et al. 2009; Reyner and Horne 2002; Horne and Reyner 2001). The purpose of the study reported here was to expand the data available on RBED by examining its hemodynamic effects during long-distance driving. To this end, we conducted a laboratory experiment using simulated monotonous driving, and recorded hemodynamic variables under double-blind counter-balanced crossover experimental conditions. As expected, the findings clearly indicated that RBED significantly changed cardiovascular variables in the direction of arousal, *i.e.*, elevation in cardiac function, as compared with the placebo-control or no drink. These results suggest that RBED has positive effects on those undertaking prolonged driving in that their cardiovascular system is in a more responsive state and this in turn may improve safety in this situation.

**Figure 3:**
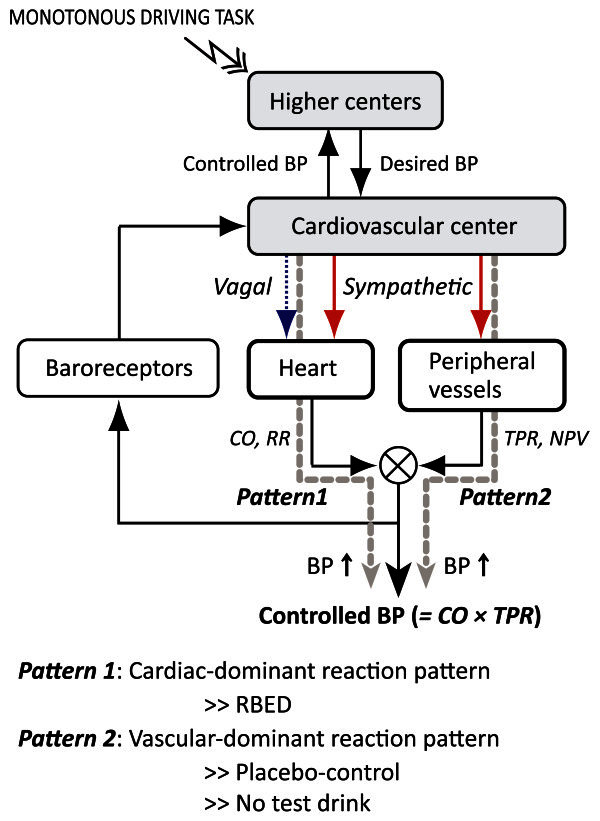
**Schematic drawing of the simplified circulatory regulation model.** Note: *BP* = blood pressure; *CO* = cardiac output; *RR* = electrocardiogram RR interval; *TPR* = total peripheral-vascular resistance; *NPV* = normalized pulse volume.

BP is generally known as the final output of the cardiovascular system through the circulatory regulation system. That is, according to the simplified circulatory regulation model (deBoer et al. 1987), BP is fed back by the heart and/or the peripheral vessels through the autonomic nervous system so as to maintain the BP at a desired level (see Figure 3). In this model, the heart modifies CO (and RR) and the peripheral vessels modify TPR (and NPV). The controlled BP is therefore calculated as “*BP* = *CO* × *TPR*”. As described above, if BP is elevated, the mechanism responsible could be described as having two patterns: one is *via* an increase in cardiac function (the cardiac-dominant reaction pattern: the rise in BP is mainly due to increased CO and decreased RR), the other is *via* an increase in vascular function (the vascular-dominant reaction pattern: the rise in BP is mainly due to increased TPR and diminished NPV). Typically, the former is observed in active coping (coping exerted under the presence of response-reinforcement contingency: it enables individuals to work on the stressful task in a successful manner), the latter in passive coping (coping exerted under the absence of response-reinforcement contingency: it forces individuals to tolerate only passively) (Sawada et al. 2002; Williams 1986; Obrist 1981). In the present study, MBP increased in all groups with time. However, the underlying mechanism of MBP elevation appeared to differ in each group. First, RBED demonstrated the cardiac-dominant reaction pattern, due to the rise in CO and decrease in RR (see Figure 3, *Pattern 1*). Second, the two control conditions (*Cont* and *ND)* exhibited the vascular-dominant reaction pattern, due to the rise in TPR and decrease in NPV (see Figure 3, *Pattern 2*), which means that vasoconstriction occurred. In fact, this passive coping is a distinctive response in monotonous driving situations (Yamakoshi et al. 2009b; Yamakoshi et al. 2009a; Yamakoshi et al. 2006), and a reaction typical to this pattern was observed in the two control conditions as mentioned above. Interestingly, however, despite the monotonous driving task, RBED triggered a change to the opposite (active coping: cardiac-dominant) pattern. This might be in part because the 80 mg (approximately 1.3 mg/kg in this study) of caffeine in RBED had a pharmacological effect on the body (specifically the heart).

In fact, the effects of caffeine alone on cardiovascular indices have been reported in many previous studies (Green and Suls 1996; Lovallo et al. 1991; MacDougall et al. 1988; France and Ditto 1988; Lane and Williams 1985; Whitsett et al. 1984; Lane 1983). First, it has been reported that caffeine and psychological stress have similar effects on cardiovascular reactivity (Lane 1983). In this study 200–250 mg of caffeine consumption was found to elevate BP and decrease RR in healthy young males and females during periods of rest and stress (MacDougall et al. 1988; Lane and Williams 1985; Lane 1983). Most notably, (Lovallo et al. 1991) examined the cardiovascular hemodynamic effects of 3.3 mg/kg caffeine plus behavioral stress in men at either low or high risk of essential hypertension. During the task there was found to be a supra-additive increase in the SBP response, which resulted from an enhanced rise in CO together with a decrease in RR, producing equivalent BP increases in both groups. Such physiological reactions are plausible as already described above using a circulatory regulation model, and our RBED findings are consistent with this result. Thus, it could be hypothesized that 80 mg caffeine in RBED, although less than half the amount of caffeine used in these previous studies, could be the main active ingredient with regard to cardiovascular behavior during prolonged, simulated, monotonous driving. However, the other ingredients of RBED, for example taurine, may have contributory effects on such reactions, as some authors have speculated (Reyner and Horne 2002; Horne and Reyner 2001; Alford et al. 2001), so this will need to be investigated, for example by including 80 mg caffeine control arms in further studies. Despite all of these considerations it is still worth bearing in mind that both RBED in combination with prolonged monotonous driving and the independent task itself produce an elevation in BP, which may have implications for long-distance drivers.

Subjective sleepiness, as assessed by KSS-J, showed a non-significant tendency to be higher in the two control conditions as compared with RBED (see Figure 2). This is at odds with previous research on RBED (Mets et al. 2011; Gershon et al. 2009; Reyner and Horne 2002; Horne and Reyner 2001). Meanwhile, the SRR as an index of driver performance showed the effectiveness of RBED relative to the two control conditions, which did not differ significantly from each other in this study. This also contradicts the findings of previous studies (Mets et al. 2011; Gershon et al. 2009; Reyner and Horne 2002; Horne and Reyner 2001). We can think of two reasons for this discrepancy. One is the relatively short driving time in this study. We used a 90-min monotonous driving task, while such tasks in other studies exceeded 120 min. The other is the difference in experimental design and purpose. In previous studies, RBED was given to sleepy subjects, *i.e.,* sleepiness was enhanced by previously undertaking a long driving task (Mets et al. 2011) or by restricting sleep to 5 h the night before (Reyner and Horne 2002; Horne and Reyner 2001). It could therefore be said that subjective sleepiness was extremely high and driving performance accordingly poor in these previous studies. Taking this into consideration, it could be hypothesized that RBED changes hemodynamic behavior relatively rapidly in the positive direction in sleepy drivers, or that the cardiac-dominant physiological response indicated in this study will have positive effects on subsequent performance. Further studies should be conducted to validate these hypotheses. In contrast to monotonous driving, it is of great interest to consider whether or not RBED could enhance driving performance and physiological behavior in scenarios such as motor racing. Racing drivers face extreme levels of physical, mental, and thermal stress (Matsumura et al. 2011; Yamakoshi et al. 2010; Yamakoshi et al. 2013), which RBED could potentially alleviate, thereby helping drivers maintain better concentration. Further investigations of this scenario will therefore be needed in a driving simulator and in real racing conditions.

One of the limitations of this study was that this test was conducted in a driving simulator environment. The development of sleepiness and the experience of monotony may differ between simulated and actual driving. The absence of actual risk in the driving simulator also differs from on-the-road driving. The effect of RBED on driving performance should therefore preferably be replicated in real situations. Secondly, with a mean age of 21.7, the population of only male drivers was relatively young. Although energy drinks are popular among this age group, it might be interesting to examine the effects of RBED in older, more experienced drivers. Thirdly, we used the same volume of test drinks in this study. However the possible effects of body weight to RBED volume ratio are likely to be significant and this needs to be explored fully in future studies. Finally, our experimental paradigm of simulated monotonous driving was relatively short. More detailed research into the relationship between cardiovascular hemodynamics and driving performance in long-distance driving should be conducted in the future.

## Conclusions

Red Bull® Energy Drink, RBED, significantly changed cardiovascular system behavior into the cardiac-dominant reaction pattern, whereas the placebo-control and ‘no drink’ subjects exhibited the expected distinctive vascular-dominant reaction pattern, in monotonous driving situations. These results suggest that RBED consumption by non-sleepy drivers before prolonged driving could help to increase arousal, leading to a safer driving status by improving concentration.

## Abbreviations

RBED: Red Bull® Energy Drink
JESS: Japanese version of the Epworth sleepiness scale
BP: Blood pressure
SBP: Systolic blood pressure
MBP: Mean blood pressure
DBP: Diastolic blood pressure
CO: Cardiac output
RR: Electrocardiogram RR interval
TPR: Total peripheral-vascular resistance
NPV: Normalized pulse volume
SRR: Standard deviation of the steering-wheel reversal rate
KSS-J: Japanese version of the Karolinska sleepiness scale.
